# Microsoft Kinect Visual and Depth Sensors for Breathing and Heart Rate Analysis

**DOI:** 10.3390/s16070996

**Published:** 2016-06-28

**Authors:** Aleš Procházka, Martin Schätz, Oldřich Vyšata, Martin Vališ

**Affiliations:** 1 Department of Computing and Control Engineering, University of Chemistry and Technology, 166 28 Prague 6, Czech Republic; Martin.Schatz@vscht.cz (M.S.); Vysatao@gmail.com (O.V.); 2 Czech Institute of Informatics, Robotics and Cybernetics, Czech Technical University, 166 36 Prague 6, Czech Republic; 3 Department of Neurology, Faculty of Medicine in Hradec Králové, Charles University, 500 05 Hradec Králové, Czech Republic; Valismar@seznam.cz

**Keywords:** MS Kinect data acquisition, image and depth sensors, computational intelligence, human–machine interaction, breathing analysis, neurological disorders, visualization, big data processing

## Abstract

This paper is devoted to a new method of using Microsoft (MS) Kinect sensors for non-contact monitoring of breathing and heart rate estimation to detect possible medical and neurological disorders. Video sequences of facial features and thorax movements are recorded by MS Kinect image, depth and infrared sensors to enable their time analysis in selected regions of interest. The proposed methodology includes the use of computational methods and functional transforms for data selection, as well as their denoising, spectral analysis and visualization, in order to determine specific biomedical features. The results that were obtained verify the correspondence between the evaluation of the breathing frequency that was obtained from the image and infrared data of the mouth area and from the thorax movement that was recorded by the depth sensor. Spectral analysis of the time evolution of the mouth area video frames was also used for heart rate estimation. Results estimated from the image and infrared data of the mouth area were compared with those obtained by contact measurements by Garmin sensors (www.garmin.com). The study proves that simple image and depth sensors can be used to efficiently record biomedical multidimensional data with sufficient accuracy to detect selected biomedical features using specific methods of computational intelligence. The achieved accuracy for non-contact detection of breathing rate was 0.26% and the accuracy of heart rate estimation was 1.47% for the infrared sensor. The following results show how video frames with depth data can be used to differentiate different kinds of breathing. The proposed method enables us to obtain and analyse data for diagnostic purposes in the home environment or during physical activities, enabling efficient human–machine interaction.

## 1. Introduction

**Figure 1:**
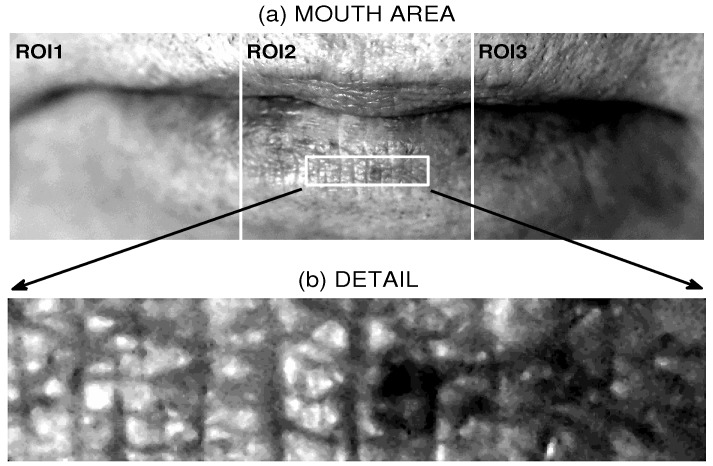
Video region used for detection of breathing frequency and heart rate presenting (**a**) the region of interest (ROI) at the mouth area and (**b**) its detail.

Recently developed computational technologies enable the use of non-contact and non-invasive systems for monitoring breathing features, detecting heart rate changes and analysing facial features as important diagnostic tools for studying neurological and sleep disorders, assessing stress and evaluating fitness level [1,2,3,4,5,6,7,8]. Figure 1 presents an example of the mouth area with the proposed use of capillaries for heart rate detection. The methodology of subtle color changes caused by blood circulation is complementary to the extraction of pulse rate from video records of fine head oscillation that accompany the cardiac cycle [9,10].

**Figure 2:**
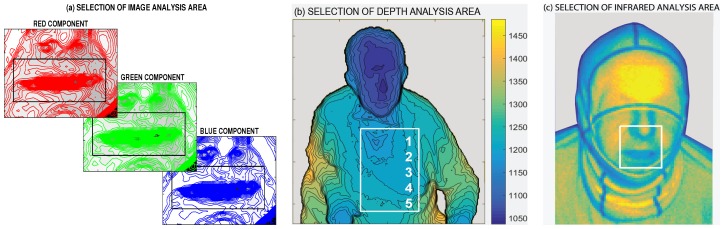
Data acquired by Microsoft (MS) Kinect presenting (**a**) red, green and blue image components; (**b**) depth sensor data; and (**c**) infrared data of a selected video frame with the area used for the time evolution of the object features.

Complex monitoring of selected biomedical features can be based on processing of data (Figure 2) recorded by Microsoft (MS) Kinect sensors [11,12,13,14,15], forming an inexpensive replacement for conventional devices. These sensors can be used to monitor breathing and heart rate changes during physical activities or sleep in order to analyze disorders by mapping chest movements during different kinds of breathing with analysis of volume or detection of neurological movement disorders and their diagnostics. In general, the use of image and depth sensors [16,17,18,19] allow 3D modelling of the chest and abdomen volume changes during breaths as an alternative to conventional spirometry with the use of video (RGB) cameras [20,21], infrared cameras [22,23,24] or Doppler multi-radar systems [25,26] in addition to ultrasonic and biomotion sensors [27,28,29].

The present paper is devoted to (i) the description of methodology for breathing and heart rate monitoring based on image, depth and infrared video sequences acquired by MS Kinect in the face and chest area; (ii) the visualization of the obtained data; and (iii) the comparison of biomedical features evaluated from data recorded by non-contact image and depth sensors.

The proposed methodology assumes the use of digital filtering methods for data noise component rejection, resampling, data fusion and spectral analysis for detecting the required biomedical features. Specific statistical methods [30,31] are applied for analysis of the obtained features. The paper presents how new technologies, data fusion of signals acquired from different sensors and specific computational intelligence methods can be used in human–machine interaction systems for data processing and extracting and analysing biomedical features.

The paper provides motivation for further studies of methods related to big data processing problems, dimensionality reduction, principal component analysis and parallel signal processing to increase the speed, accuracy and reliability of the results. Methods related to motion recognition can be further used in multichannel biomedical data processing for the detection of neurological disorders, for facial and movement analysis during rehabilitation and for motion monitoring in assisted living homes [32,33,34]. Further studies are devoted to non-intrusive sleep monitoring systems for analysis of depth video data to track a person’s chest and abdomen movements over time [35,36] for diagnosis of sleep disorders.

## 2. Methods

### 2.1. Data Acquisition

The sequence of separate images was recorded with associated time stamps indicating the video frame rate changing from 7 to 15 fps. Image resolution was 1920 by 1080 pixels while the depth and infrared camera resolution was 512 by 424 pixels. Figure 2 presents data subregions that were analysed by an MS Kinect system [13,14].

The video camera allowed following colour image components with selected results recorded at the mouth area of the individual as presented in Figure 2a in the contour form. Specific mouth detection algorithms and face recognition techniques can be used to observe this region [37,38] using different computer vision methods.

Figure 2b presents the mapping area used by the depth sensor [39] to record the distance of individual pixels in mm stored in a matrix with its size corresponding to the specified resolution. Figure 2c presents the infrared camera image. Rectangular boxes in each figure show the area used for specifying the time evolution of the object features.

Simple tests were limited by the data size that was obtained. By scaling the image data by a factor of 0.4, the 120 s record of data from the image, depth and infrared sensors occupy 1.2 GB of hard disk space. The overnight record of sleep activities requires about 250 GB of disk space and is dependent on the frame rate and resolution; specific methods of big data processing should be applied to reduce the evaluation time.

### 2.2. Data Processing

Time stamps recorded with each video frame allowed resampling of the video sequence [40,41] to a constant sampling frequency in the preprocessing stage. The final frame rate of $f_{s}=10$ fps and spline interpolation was selected to compensate sampling time changes given by technical conditions of data acquisition [42].

The sequences of image and depth maps that were acquired by the MS Kinect were analysed over the selected rectangular area of *R* rows and *S* columns that covered either the mouth or the chest area of the individual. For each matrix $D_{n}\left(i,j\right)$ including values inside the region of interest at discrete time *n*, the mean values were evaluated by the following equation:

(1) $$d\left(n\right)=\frac{1}{R\;S}\sum_{i}\sum_{j}D_{n}\left(i,j\right)\;.$$

In this way, the mean value of pixels in the selected area for each video and depth frame formed a separate time series ${\left\{d\left(n\right)\right\}}_{n=0}^{N-1}$ of length *N*. To optimize the final algorithm, the resampling mentioned above was applied to this sequence only and not to the complete image frame in this case.

Finite impulse response (FIR) filtering filtering of the selected order *M* (=40) was then applied to each obtained signal to evaluate the new sequence ${\left\{y\left(n\right)\right\}}_{n=0}^{N-1}$ using the following equation:

(2) $$y\left(n\right)=\sum_{k=0}^{M-1}b\left(k\right)\;d\left(n-k\right)\;,$$

with coefficients ${\left\{b\left(k\right)\right\}}_{k=0}^{M-1}$ defined to form a band-pass filter with cut-off frequencies $f_{1}=0.2$ Hz and $f_{2}=2$ Hz to cover the estimated frequency of breathing and heart rate and to reject all other frequency components including the mean signal value and its additional noise. Filtering was applied in both forward and reverse directions to minimize start-up and ending transients. To detect the heart rate from the depth sensor data, the infinite impulse response (IIR) band-pass filtering by the Butterworth filter of the 4th order was applied to extract frequency components in the range of 〈0.6,1.8〉 Hz.

Spectral components in each recorded frame were then evaluated by a discrete Fourier transform forming the sequence

(3) $$Y\left(k\right)=\sum_{n=0}^{N-1}x\left(n\right)\;\exp\left(-j\;k\;n\;\frac{2\;\pi }{N}\right)\;,$$

for k=0,1,⋯,N−1 related to frequency $f_{k}\;=\;\frac{k}{N}f_{s}$. To obtain the time dependence of these values, a short time Fourier transform of the selected window length was applied.

The local polynomial approximation of evaluated spectral components was then applied in two specified frequency ranges corresponding to possible frequencies of breathing ($\langle F_{1},F_{2}\rangle$ Hz) and heart rate ($\langle F_{3},F_{4}\rangle$ Hz). Extremal values are then detected in these ranges by the smoothing polynomial $g\left(x\right)\;\;=\;\;\sum_{p=0}^{P-1}c_{p}\;x^{p}$ of the selected order *P* using the least squares method to minimize summed squared differences

(4) $$S\left(c_{0},c_{1},\cdots ,c_{P-1}\right)=\sum_{k=1}^{K}{\left(g\left(f_{k}\right)-s\left(f_{k}\right)\right)}^{2}$$

between values of the smoothing function $g(f_{k})$ and *K* values of spectral components $s(f_{k})$ for frequencies $f_{k}$.

## 3. Results

**Figure 3:**
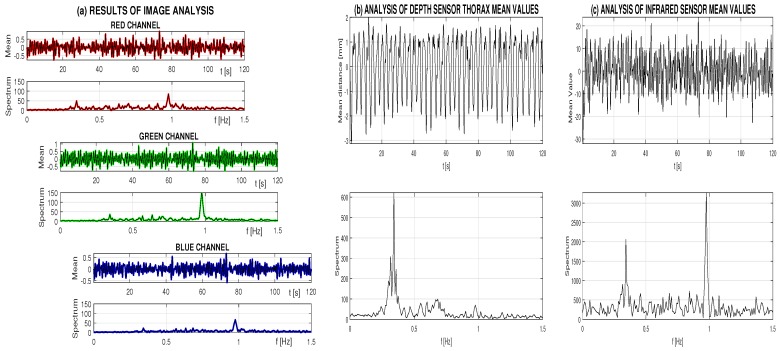
Time evolution of mean data values in selected frames with their spectra of (**a**) the red, green and blue components acquired by the image sensor; (**b**) the depth sensor data evolution with its spectrum; and (**c**) the infrared data detecting both the breathing frequency and the heart rate during a 120 s data segment.

Analysis of a selected record of 120 s of image, depth and infrared video frames in stable conditions is presented in Figure 3. The evolution of the red, green and blue mean image values in the mouth area that were selected according to Figure 1a and Figure 2a are presented in Figure 3a. Spectral analysis of these values indicates that the first dominant frequency components are approximately 0.34 Hz (representing a breathing rate of about 20.5 breaths per minute). The second dominant frequency of about 0.95 Hz (with the highest peak in the green component) represents a heart rate of about 57 bpm for this record.

The estimation of the breathing rate was confirmed by the mean thorax movement that was observed in the selected area according to Figure 2b with the resulting evolution of mean depth matrix values presented in Figure 3b. The dominant frequency peak indicates a frequency of 0.343 Hz in this case (representing 20.58 breaths per minute). Table 1 presents the comparison of these values with estimated breathing rates detected by the individual image and infrared MS Kinect sensors in areas selected according to Figure 2a,c with the associated time evolution of mean values and their spectra in Figure 3a,c. Errors related to the frequency detected by the depth sensor are in the last column of Table 1. The accuracy of image and depth sensor data analysis is better than 0.26% of the breathing rate determined by the thorax movement in this case.

The depth sensor is able to detect not only the respiratory rate but also the heart rate represented by a small peak for frequency of about 0.95 Hz (57 bpm) in this record as presented in Figure 2b. To extract this information more precisely, the band-pass filter was applied.

**Figure 4:**
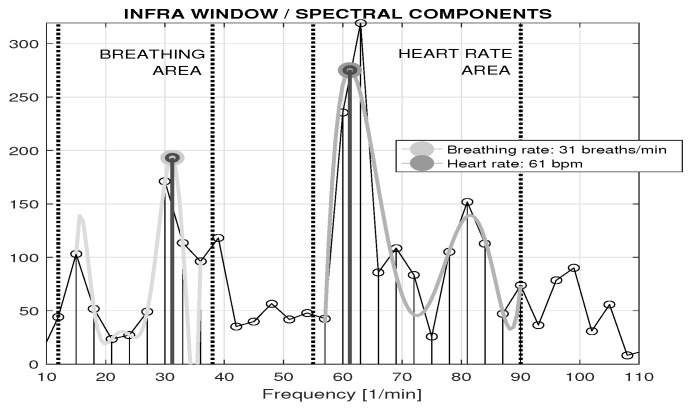
Estimation of the heart rate and breathing frequency from a 20 s infrared video sequence using mean values of the mouth ROI and showing analysis of spectral components in frequency ranges of breathing and heart rate, with extreme values detected by a local polynomial approximation.

A general methodology for the estimation of respiratory rate and heart rate is presented in Figure 4 for the spectral analysis of a 20 s infrared video sequence using mean values in the mouth region of interest (ROI). An approximation of the spectral components in the selected range using a 7th order polynomial was used to detect the frequency of the highest peak, using the mean squares method to minimize summed squared differences defined by Equation (4). The breathing rate was estimated in the selected frequency band of 〈12,38〉 breaths per minute. In a similar way, the heart rate was estimated using the local polynomial approximation in the selected frequency band of 〈55,90〉 bpm.

Analysis of the heart rate estimate based upon a 180 s infrared video sequence is presented in Table 2. Video data were recorded after physical activity following a decrease in heart rate. The region of interest (ROI) covered the mouth area according to Figure 1 and Figure 2c. Each window was 20 s in duration, and spectral analysis was performed for the whole ROI and three subregions (ROI1, ROI2, ROI3) that were selected according to Figure 1a. Results obtained by the MS Kinect non-contact infrared sensor are compared with the heart rate recorded by the Garmin heart rate sensor in the second column of Table 2. The accuracy of the heart rate estimation in the whole ROI of the infrared sensor related to the contact heart rate sensor is better than 1.5% in this case.

**Figure 5:**
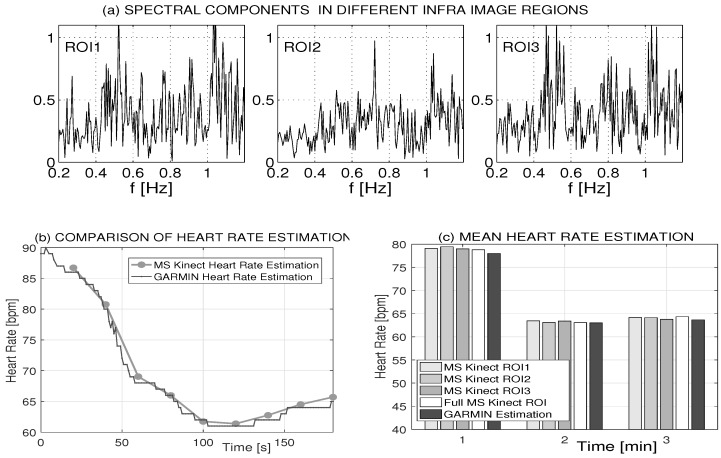
Heart rate estimation using MS Kinect infrared data and Garmin heart sensor data recorded with a sampling period of 1 s presenting (**a**) the infrared image spectral components in separate regions over a period of 180 s; (**b**) the evolution of heart rate using 20 s video sequences together with Garmin records; and (**c**) the comparison of mean heart rate values averaged over one minute segments and infrared values averaged over the whole mouth ROI and its three subregions.

Different spectral components estimated in separate subregions over the whole period of 180 s are presented in Figure 5a. While the breathing frequency can be clearly detected (because of the oral corner movement) in ROI1 and ROI3, the ROI2 covering the central part of the mouth area includes more information about the heart rate. Estimates obtained in the whole ROI and the corresponding values recorded by the Garmin sensor are presented in Figure 5b. Average heart rate values that were evaluated in each recorded minute presented in Figure 5c show very good correspondence of these observations.

**Figure 6:**
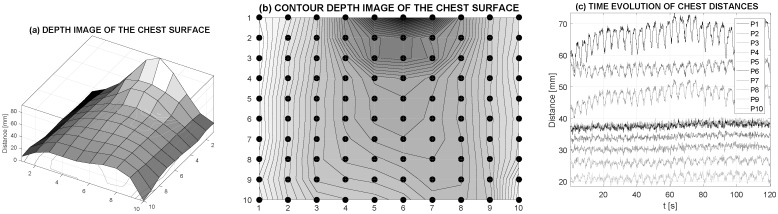
Mapping of chest movement recorded by the depth MS Kinect sensor presenting (**a**) the depth image of the chest surface in its selected area analysed on a grid with a resolution of 10 by 10 elements; (**b**) the contour depth image of the chest surface at the specified time; and (**c**) the time evolution of distances at the vertical axis of the mapped area during a 120 s data segment.

Figure 6 presents mapping of the chest movement in the selected area covered by a grid of specified density. The three-dimensional surface of the thorax that was recorded and analysed in a given time instant is presented in Figure 6a. The contour depth image of the chest with a grid of 10 by 10 values at the specified time is given in Figure 6b. Figure 6c presents the time evolution for 10 grid positions on the vertical axis of the chest showing different breathing ranges at different locations on the chest. Corresponding video animation can be used to visualize the time evolution of the thorax movement; video animation also provides the possibility to evaluate volume changes.

**Figure 7:**
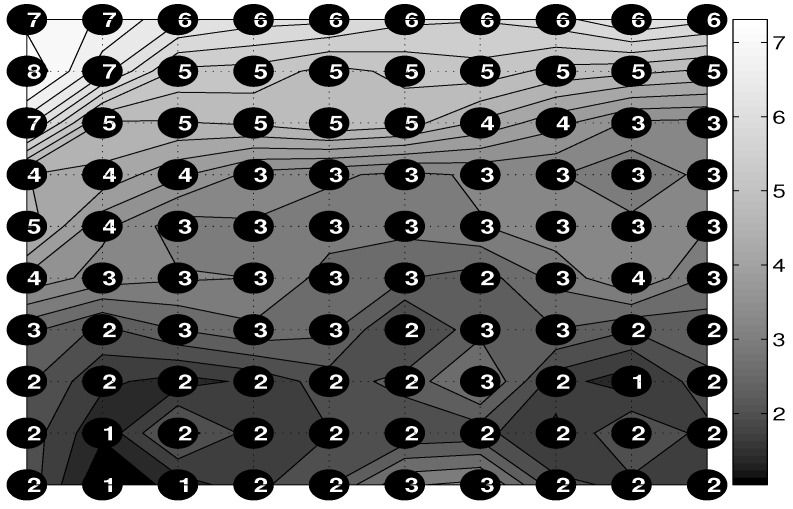
The contour map of average breathing ranges inside the selected area of the chest for the given grid density with their values (in mm) evaluated over the time range of 180 s.

The average range of chest movement in selected grid points and over the selected time of 120 s is presented in Figure 7. It is possible to observe the higher range in the upper chest area and smaller values in the abdomen area. This method can also be used for non-contact study of the symmetry of breathing movements over the chest.

**Figure 8:**
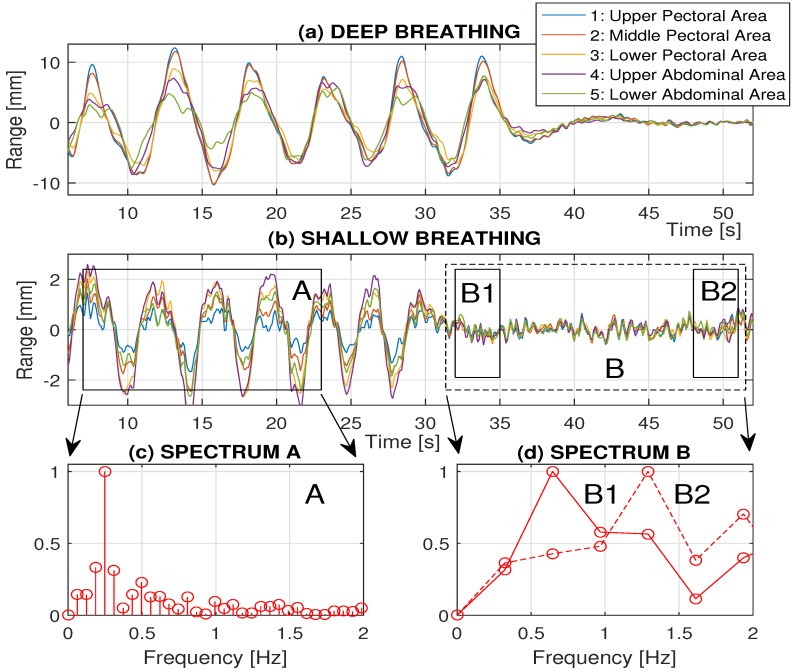
Comparison of breathing patterns in different parts of the chest (specified in Figure 2b) presenting (**a**) deep and (**b**) shallow breathing, showing interrupted breathing in the final part of each record; (**c**) associated spectral components in window A with the dominant breathing frequency of 0.27 Hz (16.2 breaths per minute); and (**d**) spectral components in subwindows B1 and B2 with the dominant heart rate frequency of 0.69 Hz (41.4 bpm) and 1.34 Hz (80.3 bpm), respectively.

Figure 8 presents a comparison of breathing patterns for deep and shallow breathing. For the deep breathing in Figure 8a, there is a dominant movement in the upper pectoral area with its range of about 16 mm, which is reduced to about 2 mm for the shallow breathing in the given record presented in Figure 8b. Abdominal movement is, on the other hand, side dominant for the shallow breathing.

Final parts of each record in Figure 8a,b present records acquired by the depth sensor during the interrupted breathing to simulate the sleep apnea While frequency components associated with the breathing disappeared, it is still possible to observe and to detect the heart rate as presented in Figure 8c,d after evaluation of spectral components of data in the middle pectoral area.

Figure 8c presents spectral components in window A with the dominant breathing frequency of 0.27 Hz (16.2 breaths per minute) in this case. Dominant frequency components in Figure 8d evaluated in subwindows B1 of the area B with the interrupted breathing points to the heart rate frequency of 0.69 Hz (41.4 bpm). During the interrupted breathing, the heart rate increases as presented for subwindow B2 pointing to the heart rate frequency of 1.34 Hz (80.3 bpm). This result shows that, for detection of sleep apnea both breathing and heart rate frequency should be observed and analyzed.

MS Kinect provides a cheap alternative to video monitoring of sleep abnomalities performed in sleep laboratories by polysomnography as the gold standard diagnostic tool. Polysomnography (PSG) records different biosignals, but it is an invasive method that may disturb natural sleep. In comparison with other efficient methods, including infrared video monitoring [24], it is possible to use MS Kinect as a cheap device to record video, depth and infrared videosequences for sleep monitoring but also as a part of assistive home technologies.

## 4. Conclusions

This paper presents the use of MS Kinect image, depth and infrared sensors for non-contact monitoring of selected biomedical signals, evaluation of breathing and heart rate using recorded video frames, and verification of the obtained results. It is assumed that estimated features obtained from data retrieved from a natural environment will increase the reliability of such observations related to the diagnosis of possible neurological disorders and the fitness level of the individual. The purpose of this approach is to replace wired sensors by distance monitoring, allowing more convenient data acquisition and analysis.

Obtained data were used for the analysis of biomedical signals recorded after specified body activity. The results show no significant difference between biomedical features obtained by different biosensors and non-contact MS Kinect technology. While breathing rate can be recorded with high reliability, non-contact heart rate detection depends upon the visibility of an individual’s blood vessels in the case of video sensor use.

Results show how the MS Kinect sensors and selected digital signal processing methods can be used to detect the heart rate and to analyse breathing patterns during different kinds of breathing. Processing of video frames acquired during interrupted breathing points to the possible use of these sensors for sleep apnea analysis as well.

The methods described here form an alternative approach to biomedical data acquisition and analysis. Developing the abilities of different biosensors with possibilities of wireless data transmission increase the importance of remote data acquisition and signal analysis using computational intelligence and information engineering in the future. This approach has a wide range of applications not only in biomedicine but also in engineering and robotics. Specific applications based on analysis of depth matrices allow gait analysis and early diagnosis of locomotor system problems.

Further research will be devoted to algorithms for more precise data acquisition and processing to detect biomedical feature changes for correct diagnosis and for proposing further appropriate treatment. It is assumed that infrared sensors will be used for non-contact analysis during sleep to detect sleep disorders.

## Figures and Tables

**Table 1:** Estimates of the respiratory frequency evaluated by Microsoft (MS) Kinect sensors in given regions of interest over a period of 120 s in stable conditions.

MS Kinect Sensor	Respiratory Rate (Breaths/min)	Error
Depth sensor	20.580	
Image sensor: red component	20.592	0.06
Image sensor: green component	20.532	0.23
Image sensor: blue component	20.532	0.23
Infrared sensor	20.526	0.26

**Table 2:** Estimates of heart rate (HR) evaluated for a 20 s window length by an MS Kinect infra sensor in the full region of interest (ROI) and its subregions in the mouth area compared with Garmin records.

Starting Time (s)	Garmin Record (bpm)	Kinect HR (bpm) Estimate				ROI Error (%)
		ROI1	ROI2	ROI3	ROI	
0	86	86.3	85.2	86.9	86.7	0.81
20	80	82.0	84.9	80.9	80.7	0.88
40	68	68.9	68.2	69.2	69.0	1.47
60	66	66.0	66.2	67.3	66.0	0.00
80	62	62.7	61.3	61.8	61.8	0.32
100	61	61.6	61.8	61.1	61.4	0.66
120	62	62.5	62.9	62.2	62.8	1.29
140	64	64.1	64.1	63.4	64.5	0.78
160	65	65.9	65.4	65.7	65.7	1.08

## Abbreviations

The following abbreviations are used in this manuscript:

ROI	region of interest
HR	heart rate
RGB	red-green-blue
FIR	finite impulse response
IIR	infinite impulse response
3D	three-dimensional
MS	Microsoft
fps	frames per second
bpm	beats per minute
